# Sensitive detection of spin-electric coupling in a Cr_3_ antiferromagnetic triangle

**DOI:** 10.1039/d5sc08012f

**Published:** 2025-12-04

**Authors:** Leonardo Tacconi, Shubham Bisht, Alberto Cini, Mauro Perfetti, Tomas Orlando, Maria Fittipaldi, Michael Shatruk, Roberta Sessoli

**Affiliations:** a Dipartimento di Chimica “U. Schiff”, Università degli Studi di Firenze Sesto Fiorentino Firenze I-50019 Italy roberta.sessoli@unifi.it; b Department of Chemistry and Biochemistry, Florida State University Tallahassee FL 32306 USA shatruk@chem.fsu.edu; c Dipartimento di Fisica e Astronomia, Università degli Studi di Firenze Sesto Fiorentino Firenze I-50019 Italy maria.fittipaldi@unifi.it; d Consorzio Interuniversitario Nazionale per la Scienza e Tecnologia Dei Materiali Firenze I-50121 Italy; e National High Magnetic Field Laboratory Tallahassee FL 32310 USA

## Abstract

Molecular antiferromagnetic triangles are a convenient platform to study the effect of an electric field on the magnetic exchange interactions. However, such effects are typically hard to detect, especially in systems with weak spin–orbit coupling. In this work, an asymmetric µ_3_-oxo-centered Cr_3_ triangle was synthesized and structurally characterized as a non-centrosymmetric molecular crystal suitable for probing Spin Electric Coupling (SEC). A combination of single-crystal magnetometry, cantilever torque magnetometry, and continuous-wave electron paramagnetic resonance (EPR) allowed precise determination of the spin Hamiltonian parameters, including the weak Dzyaloshinskii–Moriya interaction. Electric-field-modulated EPR (EFM-EPR) experiments provided the first direct observation of SEC in a Cr^III^-based complex, revealing measurable electric-field effects on the single-ion 
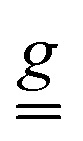
 tensor and setting an upper bound for the SEC influence on magnetic exchange interactions. These findings demonstrate the exceptional sensitivity of EFM-EPR spectroscopy for quantifying SEC and highlight the crucial role of molecular symmetry and ligand environment in enabling electric control of spin states, thus advancing the rational design of molecular systems for quantum technologies.

## Abstract 2

The high sensitivity of EPR spectroscopy under modulated electric field applied to a non-centrosymmetric Cr_3_ spin-frustrated triangle reveals weak but quantifiable spin–electric effects, highlighting how ligand choice governs spin–electric coupling.

## Graphical abstract

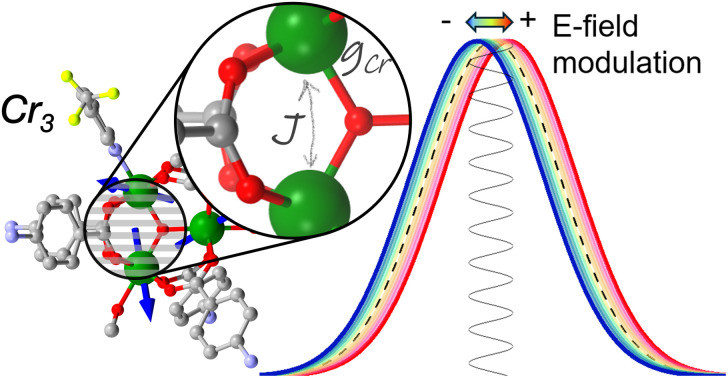

## Introduction

Spins in molecules are a potential resource for quantum technologies.^1–3^ They can act as quantum sensors of magnetic fields or as active units (qubits) in quantum information processing. If properly designed, they can exhibit remarkable quantum coherence and can be integrated into more complex architectures. Individual spin addressability remains critical if probed with magnetic fields. Conversely, electric fields can be confined at the level of a single molecule and have been employed to address molecular spins in nanojunctions^4^ or under a scanning probe tip.^5^

Electric field control of the magnetization is a widely investigated phenomenon in extended inorganic lattices,^6–9^ but less explored in molecular materials.^10^ In the latter, we can distinguish two main research lines, reflecting the different mechanisms that lead to spin-electric coupling (SEC). The first is the electric field control of single-ion magnetic anisotropy, which can be detected as a shift in the resonance field in continuous-wave Electron Paramagnetic Resonance (EPR) or the accumulation of a phase in pulsed EPR experiments.^11–16^ Additionally, spin transitions can be coherently promoted by the electric field, and the effects are enhanced in piezo-^17^ and ferroelectric materials.^18^ Spin–orbit coupling (SOC) plays a crucial role in this context, and several useful correlations with the molecular structure have been recently established.^15,19,20^ The second line, more relevant here, is the electric field control of systems with active exchange interactions between spins.^21–24^ Optimizing such an effect could provide a pathway to scalable quantum architectures based on molecular spins, as proposed by Kane for spins in semiconductors.^25^

A fascinating platform for investigating the effect of an electric field on magnetic exchange is formed by antiferromagnetic triangles of half-integer spins. Spin frustration results in two degenerate doublet ground states in the case of strict three-fold symmetry.^26^ The degeneracy can be removed by coupling with vibrations, *i.e.*, static or dynamic Jahn–Teller effect,^27^ by SOC-mediated antisymmetric or Dzyaloshinskii–Moriya (DM) exchange interaction,^28^ or by an electric field.^29,30^ EPR experiments with electric field pulses superimposed on the sequence of the electromagnetic radiation pulses have detected SEC effects in the spin echo of Fe^III^_3_ (ref. 22) and Cu^II^_3_ triangles.^12^ Conversely, no effect was detected for Cr^III^_3_ triangles,^22^ and the absence was attributed to the small single-ion magnetic anisotropy and very weak DM interactions compared to those observed for Fe^III^_3_.^22^

CW-EPR spectroscopy with electric field modulation (EFM-EPR), instead of the conventional magnetic field modulation, constitutes a relatively underutilized yet powerful tool for investigating the SEC phenomenon in single crystals, enabling a detailed analysis of the SEC anisotropy.^16,21,23,31,32^ In fact, EFM-EPR retains the full spectroscopic potential of EPR by providing informative spectral line shapes at each orientation. However, the detection of the linear effect requires the use of non-centrosymmetric crystals. We stress here that the use of EFM-EPR associated with first-order detection is possible only for samples that lack global inversion symmetry. The currents in the transmission line that charge the capacitor also generate an oscillating magnetic field,^24^ which, if overlooked, may lead to data being misinterpreted as an EFM-EPR signal from a randomly oriented powder specimen.^33^

**Fig. 1:**
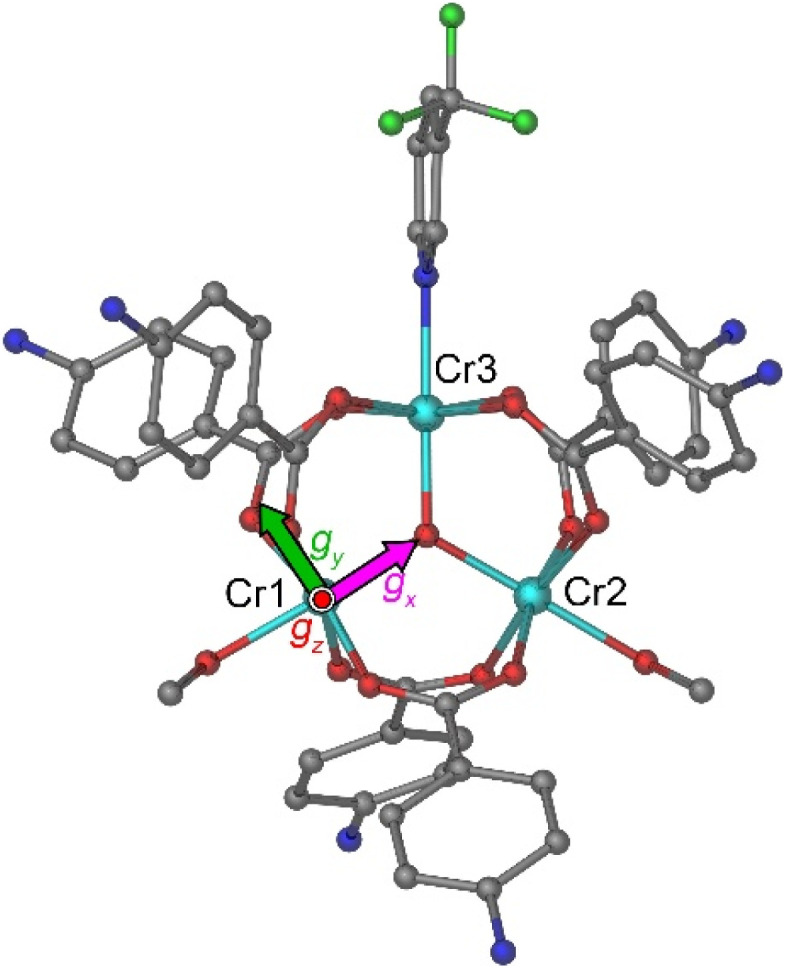
The trinuclear oxo-bridged cation in the crystal structure of 1 where the single-ion *g* reference frame is superimposed on Cr1, with the unique axis *x* directed towards the µ_3_-O atom. The H atoms are omitted for clarity. Color scheme: Cr = cyan, F = green, O = red, N = blue, C = gray.

In this study, we demonstrate the exceptional sensitivity of the EFM-EPR technique by investigating SEC in the elusive case of Cr^III^_3_, which did not show any effect in pulsed EPR experiments.^22^ To that end, we have synthesized an asymmetric µ_3_-oxo-centered triangle where six 4-aminobenzoate bridges connect the Cr^III^ ions. The terminal ligands are two methanol molecules and one 3-trifluoromethylpyridine (Fig. 1). A combination of single-crystal magnetometry (both standard and torque) and EPR experiments provided an accurate quantification of the Spin Hamiltonian parameters, including the very weak DM interaction. Single-crystal CW EPR spectra under electric field modulation were acquired and simulated, providing the first evidence of SEC in a Cr^III^-based molecular material. Our findings highlight EFM-EPR spectroscopy as a key tool for advancing understanding of SEC mechanisms and for identifying strategies to exploit it for quantum applications based on molecular spins.

## Results and discussion

All details on materials and methods are available in the SI.

### Synthesis

Complex [Cr_3_(µ_3_-O)(µ_2_-4-H_3_NBzO)_6_(3-CF_3_py)(MeOH)_2_](NO_3_)_7_ (1) was synthesized by a reaction between Cr(NO_3_)_3_ and 4-aminobenzoic acid (4-H_3_NBzOH) in MeOH, followed by addition of 3-trifluoromethylpyridine (3-CF_3_py). Performing the second step at room temperature resulted in a mixture of two types of crystals, corresponding to [Cr_3_(µ_3_-O)(µ_2_-4-H_3_NBzO)_6_(MeOH)_3_](NO_3_)_7_ (1′), previously reported by Zaworotko *et al.*,^34^ and complex 1, reported here for the first time and formed by replacement of one of the MeOH ligands in 1′ by 3-CF_3_py. Crystals of 1′ were extremely hygroscopic, while the crystals of 1 appeared more stable when exposed to air. Performing the reaction under reflux forced more complete replacement of the MeOH ligand, leading to the isolation of pure 1 in good yield (60%). Crystallization of 1 was achieved by vapor diffusion of Et_2_O into the methanolic solution of the complex. The identity of the complex was established by single-crystal X-ray diffraction (see below). The elemental analysis showed that the powder sample absorbed water upon exposure to air, to yield the bulk product as 1·6H_2_O.

The FT-IR spectroscopy revealed a characteristic peak at 630 cm^−1^, corresponding to an asymmetric vibration of the Cr_3_O triangle unit,^35,36^ as well as peaks at 1540 cm^−1^ and 1398 cm^−1^ assigned to the asymmetric and symmetric vibrational modes of the carboxylate ligands (Fig. S1). The energy difference of 142 cm^−1^ between the carboxylate vibrations is smaller than that reported for sodium benzoate (186 cm^−1^), as expected for the bidentate bridging mode of carboxylates in 1.^36,37^ TGA analysis showed an initial mass loss of ∼8%, which can be attributed to the loss of a single 3-CF_3_py ligand or the removal of crystallization solvent. The mass loss reaches 25% at 170 °C, followed by the decomposition of the complex at higher temperatures (Fig. S2).

### Crystal structure

Complex 1 crystallizes in the polar space group *Cc*, with four formula units per monoclinic cell. The asymmetric unit contains one [Cr_3_(µ_3_-O)(µ_2_-4-NH_3_BzO)_6_(3-CF_3_py)(MeOH)_2_]^7+^ cation, seven NO_3_^−^ anions, and one MeOH solvate molecule. The coordination environment of each Cr^III^ ion is a distorted octahedron (Fig. 1), with the equatorial plane formed by four O atoms of neutral zwitterionic η^1^:η^1^-µ_2_-4-NH_3_BzO ligands and the axial positions occupied by the µ_3_-bridging oxide anion in the *endo*-site and in the *exo*-site either a MeOHfor Cr(1) and Cr(2) or a 3-CF_3_py molecule for Cr(3). In contrast to the previously reported structure of 1′, with the high-symmetry space group *R*3*m*, the crystal structure of 1 exhibits lower symmetry. The substitution of 3-CF_3_py for one of the MeOH molecules results in the loss of the 3-fold rotation axis and the mirror plane. The majority of complexes with the Cr_3_(µ_3_-O) triangle were reported with equivalent ligands in the axial *exo*-site,^35^ while only a few such complexes with different *exo*-ligands are known.^34,38^ Our work demonstrates that complex 1 can be formed as a pure compound in good yield by forcing the replacement of one of the MeOH molecules with the stronger pyridine-based ligand.

**Fig. 2:**
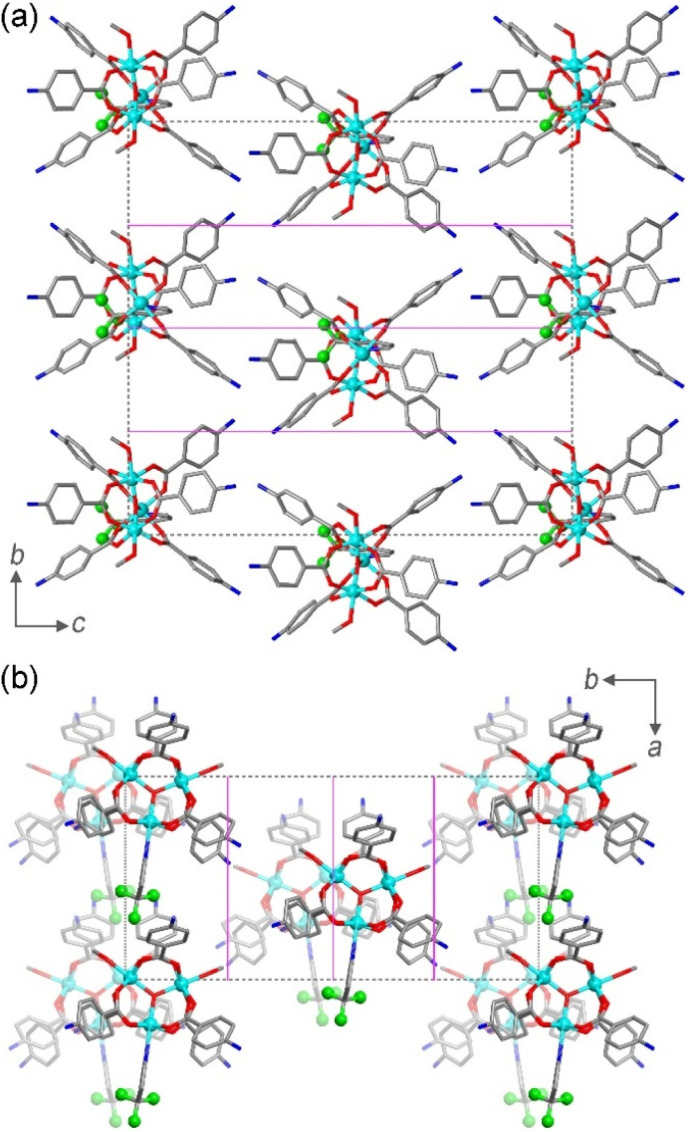
The arrangement of the trinuclear cations in the crystal packing of 1 viewed down the [100] (a) and the [001] (b) directions. The anions, solvate molecules, and H atoms are omitted for clarity. Color scheme: Cr = cyan, F = green, O = red, N = blue, C = gray. The unit cell is indicated with dashed gray lines. The magenta lines in both panels indicate the planes corresponding to the *c*-glide symmetry operation. In panel (b), this *c*-glide operation relates the molecules that appear in the front to the faded molecules that appear in the back.

The low symmetry of the molecule leads to different distances between the metal centers. The Cr(1)–Cr(2) distance (3.301(1) Å) between the MeOH-coordinated Cr^III^ ions is longer than the Cr(1)–Cr(3) and Cr(2)–Cr(3) distances to the 3-CF_3_py-coordinated Cr^III^ ion (3.279(1) Å and 3.289(1) Å, respectively). The lack of equilateral geometry in the central triangle also leads to slightly different Cr–O–Cr angles at the µ_3_-bridging oxide (Table S2). The Cr–(µ_3_-O) bonds are 1.908(4) Å, 1.894(4) Å, and 1.896(4) Å for the Cr(1), Cr(2), and Cr(3) sites, respectively, while the Cr–O_MeOH_ bonds, 2.048(4) and 2.078(5) Å, are notably shorter than the Cr–N_pyr_ bond of 2.111(5) Å. The Cr–O_BzO_ distances to the zwitterionic 4-H_3_NBzO ligands are similar for all three metal sites and vary from 1.927(5) to 2.000(2) Å. An examination of the crystal packing reveals that the two magnetically non-equivalent Cr_3_ triangles, related by a *c*-glide mirror plane perpendicular to the *b* axis, are almost coplanar, with the dihedral angle of 9.64°. The plane of each triangle is tilted by 10.64° relative to the *ab* lattice plane (Fig. 2a). When the structure is viewed approximately perpendicular to the triangular plane, down the [001] direction, it can be seen that the triangles are oriented in the same direction (Fig. 2b), with the imaginary line that bisects the Cr(1)–Cr(3)–Cr(2) angle passing at ∼19° relative to the *a* axis.

### Magnetic properties

The magnetic properties of oxo-bridged chromium triangles can be derived from the following Hamiltonian for three *s* = 3/2 spins:1
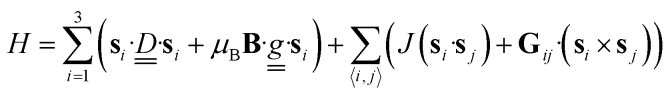


**Fig. 3:**
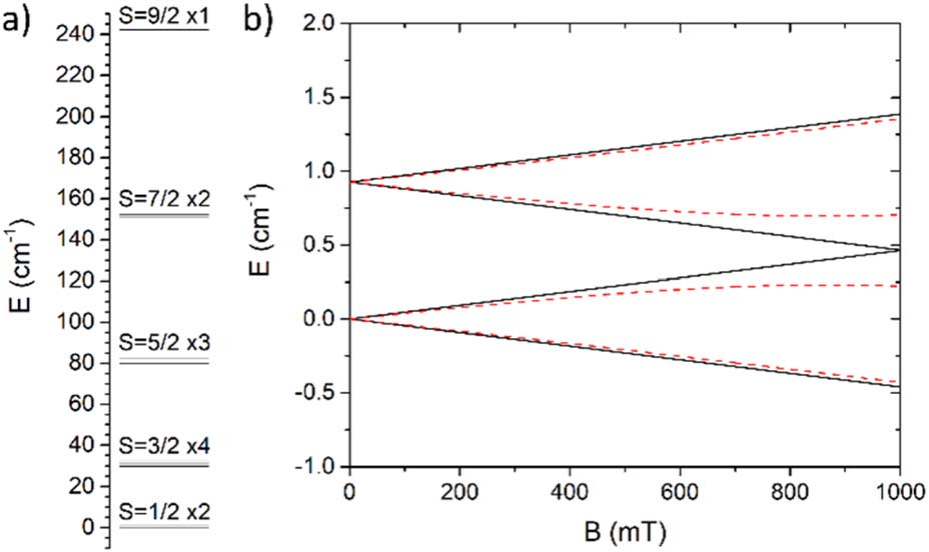
(a) Ladder of the spin levels and their degeneracy computed with the values of magnetic parameters extracted from the magnetometry data. (b) Zeeman diagram of the doublet states with the magnetic field applied perpendicular (black solid lines) and parallel (red broken lines) to the Cr_3_ plane.

The first term represents the single-ion contribution, including the zero-field splitting (ZFS) and the Zeeman interaction, while the second term accounts for exchange interactions, divided into isotropic (Heisenberg) and antisymmetric (Dzyaloshinskii–Moriya, DM) components. The anisotropic (or symmetric) part of the interactions, due to dipolar coupling, has been neglected for simplicity, as it does not affect the phenomena investigated here (see below). In oxo-bridged Cr^III^ triangles, the Heisenberg term is dominant,^39–41^ whereas ZFS and DM interactions are small perturbations linked to the spin–orbit coupling (SOC), which is largely quenched in Cr^III^. In the case of antiferromagnetic (AFM) interactions, a spin-level diagram with high degeneracy is expected, as shown in Fig. 3a.

The single ion ZFS tensor 
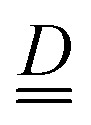
 can be estimated from the anisotropy of the 
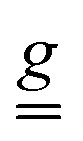
 tensor (eqn (2)), where k denotes each of the principal magnetic axes, *g*_e_ ≈ 2.0023 is the free-electron *g* factor, and *λ* = 91 cm^−1^ is the SOC constant for Cr^III^:^42^2
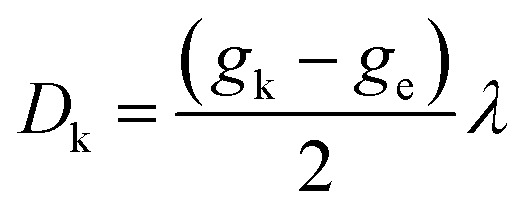


The three orthogonal components of the DM vector **G** follow general symmetry constraints. In systems possessing a *C*_3_ rotation axis, the relation *G*_*Z*_ ≫ *G*_*X*_ = *G*_*Y*_ ≃ 0 stands, where the *Z* molecular magnetic axis is parallel to *C*_3_ axis.^40^

While DC magnetometry on powder samples delivers a good estimation of the magnitude of the Heisenberg interaction,^39,43–45^ an insight into the anisotropic terms of the Hamiltonian requires single-crystal measurements. The magnetic properties of 1 were investigated by DC magnetometry on both powder (Fig. S3) and single crystal samples (Fig. S4 and S5). The magnetic field applied along two different crystallographic orientations, namely the c* and *a* axes, allowed probing the magnetic response of the molecules approximately perpendicular and parallel to the Cr_3_ triangle plane, respectively, as visually represented in Fig. 2.

The experimental *χT vs. T* curves are shown in Fig. S4. For both orientations, the room temperature value is ≈ 4.33 emu K mol^−1^, which is significantly smaller than the value expected for three uncoupled Cr^III^ ions (5.625 emu K mol^−1^) and consistent with strong antiferromagnetic (AFM) exchange interactions. Upon lowering the temperature, the value of *χT* decreases similarly for both orientations, suggesting that the ZFS and DM interactions must be considered perturbations of the AFM exchange. At 2 K, the experimental *χT* is 3% higher along the c* axis than along the *a* axis (0.393 *vs.* 0.382 emu K mol^−1^), indicative of a weak easy-axis magnetic anisotropy of the triangle. The field-dependent magnetization isotherms, shown in Fig. S5, reveal similar magnetization values along both directions, again indicating a weak easy-axis anisotropy. The maximum value obtained at 2 K and 5 T in both cases is around 0.95 *µ*_B_, in agreement with the *S* = 1/2 ground state of the spin-frustrated triangle.^46^

Powder DC magnetometry data allowed for the estimation of the average *J* value, whereas single crystal data revealed a magnetic anisotropy, which is determined by both the asymmetry in the *J* values and the DM interaction. The best fit of the DC magnetic data (Fig. S3) was obtained considering an isosceles AFM-coupled triangle with *J*_13_ = *J*_23_ = 20.0(1) cm^−1^ and *J*_12_ = 20.4(2) cm^−1^, based on the different ligand environment around the Cr^III^ ions (Fig. 1). This results in an energy gap of 30.6 cm^−1^ between the almost degenerate ground doublets and the first excited state with total spin *S* = 3/2.

Whilst single-crystal magnetic measurements demonstrate the presence of a certain magnetic anisotropy in the sample, they do not allow for disentangling the contributions from *g*-anisotropy and DM interaction. One of the primary techniques for accessing such information is cantilever torque magnetometry (CTM).^47^ It has been intensively used to reveal and understand the magnetic anisotropy in 3d^48,49^ and 4f^50–52^ metal complexes, but its application in molecular triangles has been poorly explored,^53,54^ and DM interactions have not been addressed.

**Fig. 4:**
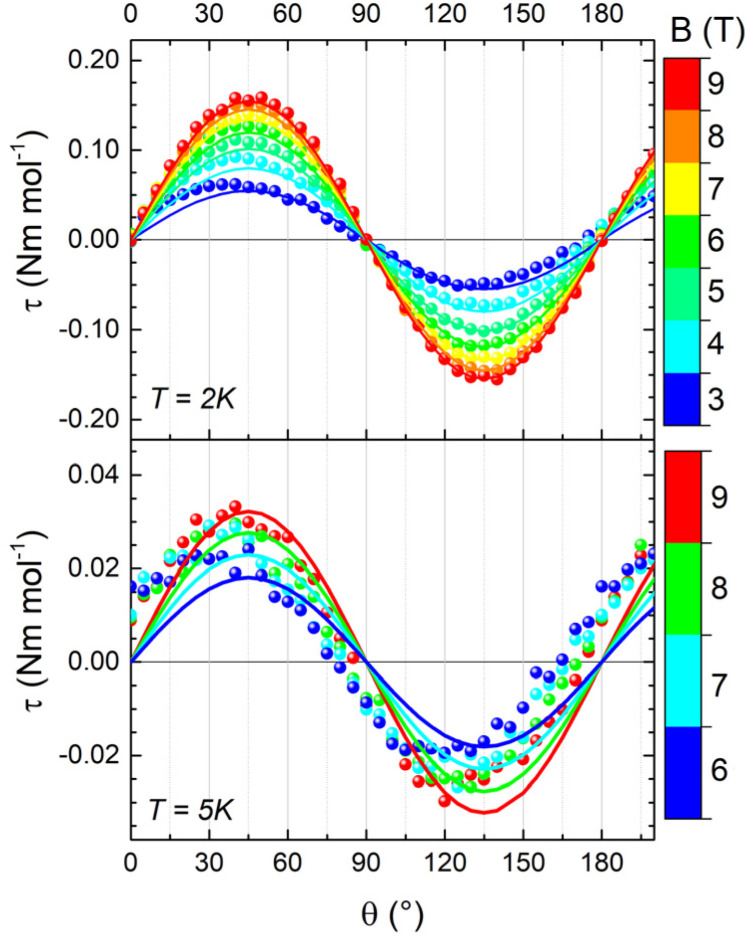
Experimental (dots) and simulated (lines) cantilever torque magnetometry curves acquired on 1 during rotation 1 at 2 K (top) and 5 K (bottom) and different magnetic fields. **B** is parallel to c* for *θ* = 0° and to **b** for *θ* = 90°. The simulated curves were calculated considering both an anisotropy of the *g* factor and the anisotropy induced by the DM interaction, as discussed in text.

We performed two different rotations of the sample (depicted in Fig. S6) around the *a* and c* axes, thus probing the triangle plane-to-axis magnetic anisotropy and the in-plane magnetic anisotropy, respectively. The results of the first rotation are shown in Fig. 4. At 2 K and 9 T, the phase of the curve unambiguously confirms the easy-axis anisotropy (*i.e.* the pseudo-*C*_3_ axis of the triangle has a larger magnetization than the plane). However, even in these extreme experimental conditions, the signal intensity is minute, suggesting a weak magnetic anisotropy. Upon increasing the temperature to 5 K, the signal rapidly diminished, hindering the acquisition of additional curves at fields below 6 T or at higher temperatures. Conversely, during the second rotation, the very weak signal could only be detected at 2 K and 9 T (see Fig. S7), thereby impeding further investigations. The lack of 120° periodicity is consistent with our model, which predicts isotropy in the plane, even for non-uniform values of the isotropic exchange constants. The 180° periodicity and the weakness of the signal in this rotation can be attributed to a minor misalignment of the crystal.

To understand the origin of such anisotropy, we simulated the experimental measurements using the Hamiltonian in eqn (1), accounting for the presence of two magnetically inequivalent molecules within the crystal. Axial 
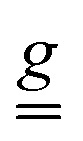
 and 
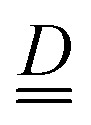
 tensors for the single-ion *s* = 3/2 were assumed with identical orientations. Their unique axis, defined as *x*, was fixed along the unique axis of the octahedron, which, for 1, corresponds to the Cr–O(µ_3_) bond. The *z* magnetic axis was oriented perpendicular to the triangle plane, while *y* completed the Cartesian tern, as indicated in Fig. 1. Under these conditions, for Cr^III^ ions, the relations *g*_*x*_ ≠ *g*_*y*_ = *g*_*z*_ and, as derived from eqn (2), *D*_*x*_ ≠ *D*_*y*_ = *D*_*z*_ stand. The Euler angles describing the rotation from the molecular to the tensor frames, *R̂*_M–T_, and from crystallographic to molecular frames, *R̂*_C–M_, are reported in Tables S2 and S3. A visual representation of the magnetic reference frames in the two inequivalent triangles is depicted in Fig. S8.

In the temperature range of CTM measurements, only the almost degenerate total spin *S* = 1/2 states are populated. As the ZFS and dipolar interactions exclusively affect excited states with higher spin multiplicity, the torque signal must arise from either the DM interaction and/or the anisotropy of the *g* factor. Due to the pseudo-trigonal symmetry of the complex, we considered *G*_*Z*_ as the dominant parameter and imposed *G*_*X*_ = *G*_*Y*_ = 0. This assumption leads to only two parameters as possible sources of magnetic anisotropy: *G*_*Z*_ and the axis-plane anisotropy of the single-ion *g* factor, indicated as δ*g* = *g*_*z*,*y*_ − *g*_*x*_.

We recall that in the case of the AFM triangles, DM interactions tend to orient the in-plane component of the spins in a toroidal arrangement, whose winding depends on the sign of *G*_*Z*_. This reduces the response to the in-plane magnetic field, *i.e.*, the effective in-plane *g* is reduced, while the out-of-plane component is practically unaffected. Additionally, the extent of deviation from the equilateral triangle is crucial in the presence of DM interaction, as the effect of DM on lowering the effective *g* value in the plane of the triangle is strongly reduced as the ratio δ*J*/*G*_*Z*_ increases, *i.e.*, if different *J* values lift the degeneracy of the two ground-state doublets.

Interestingly, *g*-anisotropy and DM interactions induce significantly different dependencies of the overall magnetic anisotropy on field and temperature – *i.e.*, practically independent for the former – allowing an accurate disentanglement of the two contributions. Therefore, we attempted two fit procedures, fixing one of the two parameters to zero. The first fit procedure (*G*_*Z*_ = 0, δ*g* = 0.012, Fig. S9) did not reproduce the in-field evolution of the signal at 2 K and its thermal evolution, resulting in a significant overestimation of the magnetic anisotropy at 5 K. The second fit procedure (δ*g* = 0, *G*_*Z*_ = 0.07 cm^−1^, Fig. S10) showed better congruence with the in-field and temperature evolutions of the signal, even though the magnetic anisotropy at 5 K remained underestimated. Consequently, both factors were considered, leading to the best parameter combination of δ*g* = 0.0013(3) and *G*_*Z*_ = 0.067(3) cm^−1^. This model accurately reproduces both the thermal and in-field evolutions of the signal, as shown in Fig. 4. The spin state energy ladder and the Zeeman diagrams computed with these parameters are shown in Fig. 3, and simulations of the DC properties are shown in Fig. S11 snd S12. The inclusion of the dipolar term in the interaction has no sizeable effect on the magnetic properties (see Fig. S13).

### Electron paramagnetic resonance

**Fig. 5:**
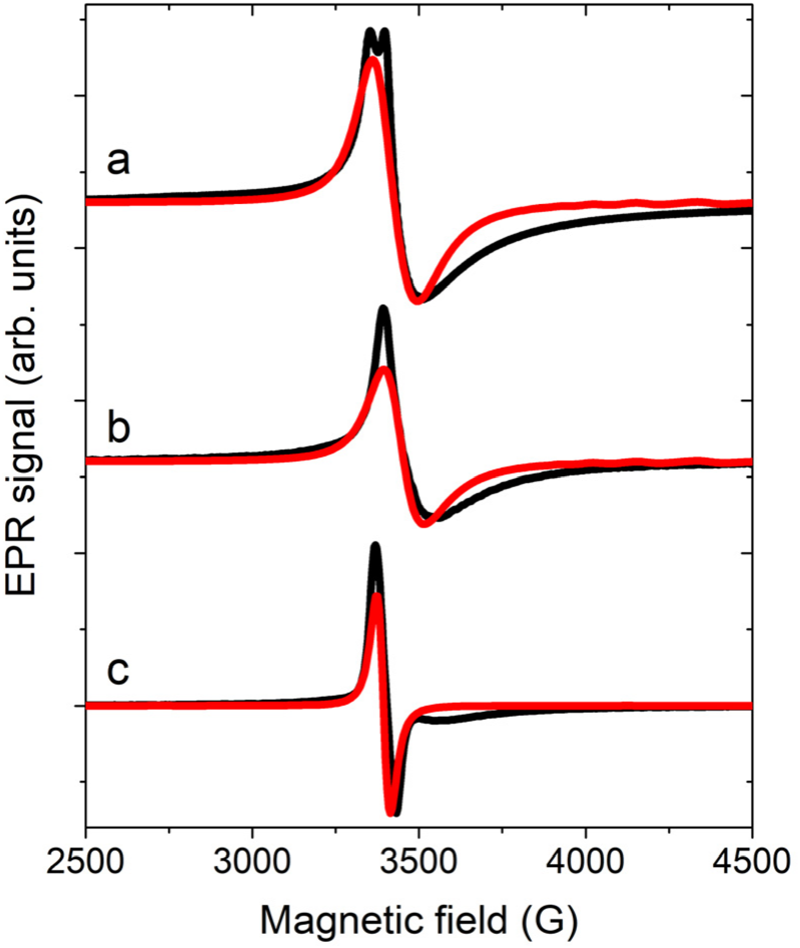
EPR spectra acquired at 5 K (black line) on a powder sample (a) and on the single crystal of 1 with **a**//**B**_**0**_ (b) and c*//**B**_**0**_ (c). Red lines are the corresponding simulations.

EPR spectroscopy is a powerful tool for determining *g* anisotropy and, in some cases, the DM interaction, even when investigating polycrystalline samples.^40,41,55,56^ X-band EPR spectra on the powder and single crystal of 1 at 5 K are reported in Fig. 5. The powder spectrum shows an almost single resonance with a linewidth of approximately 50 G and a small splitting, indicating that the parallel and perpendicular *g* values should be very similar. Moreover, the lineshape is asymmetric, a feature reported also for a similar Cr_3_ compound.^44^ The same feature is encountered in the single-crystal spectrum for **a**//**B**_**0**_*,* while for c*//**B**_**0**_ the line becomes narrower and more symmetric. The complete angular dependence of the EPR spectra is reported in Fig. S14. The asymmetric shape is quite common in frustrated AFM triangles exhibiting DM interactions, as the distribution of *J* values strongly impacts the resonance field when the field is in the plane of the triangle.^39–41,43,45,55–57^

**Fig. 6:**
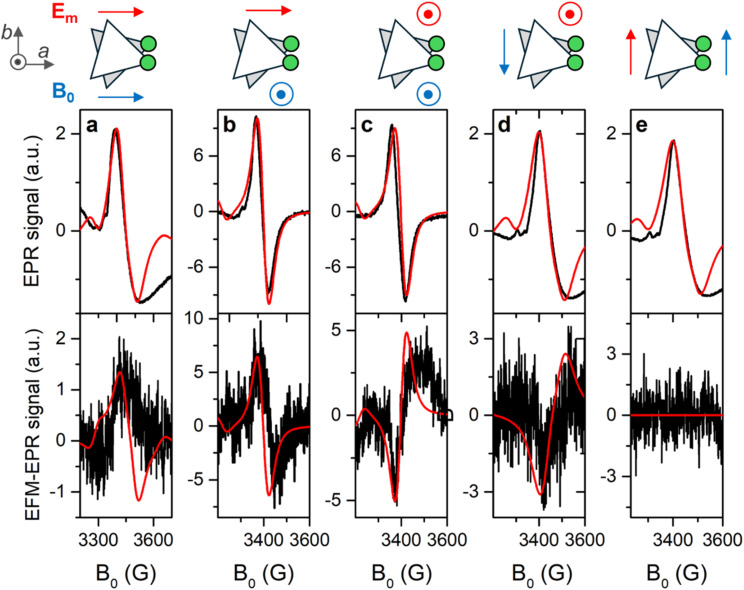
EPR spectra (top panels) and corresponding EFM-EPR spectra (bottom panels) acquired on a single crystal of 1 at 15 K in different experimental configurations as indicated in the schemes on top of each column (a–e), where the 3-CF_3_py-coordinated Cr(3) site is shown in green. The EFM-EPR spectra intensities are rescaled by the number of acquisitions *n* (*n* = 11, 4, 9, 10, and 6, respectively), necessary to achieve a reasonable signal-to-noise ratio. The red lines represent simulations as described in the text.

To simulate the spectra, we considered the model employed for CTM simulations with *g*_*y*,*z*_ = 1.976(1) and *g*_*x*_ = 1.9740(1), which led to a *D* = 3/2 *D*_*x*_ = 0.09 cm^−1^ (see eqn (2)). Concerning the exchange interaction, the isosceles model was considered with *J*_13_ = *J*_23_ = *J* = 20 cm^−1^ and *J*_12_ = *J* + δ*J*. The DM interaction with *G*_*Z*_ = 0.067 cm^−1^ was also introduced, consistent with the results of the CTM analysis. In contrast to the choice reported in the earlier work,^44^ where empirical distributions of δ*J* and/or *G*_*Z*_ values were considered to simulate the asymmetric shape, we employed only a Gaussian distribution of δ*J* values having a center at 1.10(2) cm^−1^ and *σ*_δ*J*_ = 1.10(5) cm^−1^ (see Fig. S15). These values are compatible within errors with those extracted from magnetic measurements. This model enabled us to simulate the EPR spectra measured at 5 K (Fig. 5) and those measured at 15 K, which served as a reference for the EFM-EPR measurements (Fig. 6, top panels), even though the broadening at high fields was not fully reproduced.

### EFM-EPR

The EFM-EPR technique differs from the standard EPR spectroscopy in the field modulation used to acquire the spectrum. In place of the standard magnetic field modulation, an oscillating electric field *E*(*t*) = *E*_m_cos(*ωt*) with *E*_m_ = 60 kV m^−1^ and *ω* = 2π·30 kHz is applied. This was achieved by employing a home-made setup described in SI. By employing phase-sensitive detection, a derivative signal may appear if the electric field shifts the resonance frequency of a quantity that is sufficiently small compared to the peak's intrinsic linewidth. On the other hand, an absorption-like EFM-EPR signal is detected if the oscillating electric field modifies the transition probability.

To address the anisotropy of the SEC, the EPR and EFM-EPR spectra were acquired on an oriented single crystal at 15 K for different mutual orientations between the crystal axes, the magnetic field **B**_**0**_, and the electric field **E**_**m**_. In Fig. 6a, the standard EPR spectrum acquired with the *a* axis parallel to **B**_**0**_ is reported (top panel), together with the corresponding EFM-EPR spectrum acquired with **E**_**m**_ parallel to the *a* axis (bottom panel). The latter has the same phase and center as the EPR spectrum. The phase changes sign if the direction of **E**_**m**_ is reversed (Fig. S16), as expected in a noncentrosymmetric crystal. An EFM-EPR signal is also observed rotating the sample holder by 90°, such that the *a* axis is still along **E**_**m**_, but the c* axis is along **B**_**0**_ (Fig. 6b). Moreover, a EFM-EPR signal is also clearly detected for c*//**E**_**m**_ with either c*//**B**_**0**_ (Fig. 6c) or −**b**//**B**_**0**_ (Fig. 6d). When **b**//**E**_**m**_ the EFM-EPR signal is within the noise level (Fig. 6e), as expected by symmetry reasons (**E**_**m**_ perpendicular to the mirror plane).

To understand the origin of the SEC effect and estimate its intensity, a phenomenological model was applied, starting from the one used to simulate the EPR spectra and complementing the Hamiltonian in eqn (1) with terms accounting for the effect of the **E**_**m**_ field as a perturbation on either the 
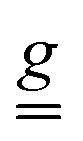
 tensor or the exchange interaction *J*. For simplicity, we have not considered a SEC on the single-ion ZFS, as we have verified that it does not substantially affect the intensity of the EFM-EPR signal.

The Δ*g* variation for each Cr^III^ center induced by the applied **E**_**m**_ results in

3 *g*_*i*_(**E**_**m**_//**j**) = *g*_*i*_ + Δ*g*_*i*,*j*_ = *g*_*i*_ + *E_j_T*_*j*,*i*_

where *g*_*i*_ represents the unperturbed principal value of the Cr 
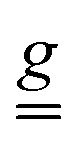
 tensor for *i* = *x*, *y*, *z*, and *j* varies over the laboratory axes (**X**, **Y**, **Z**) with **X**//**a** and **Z**//c*, while *T*_*j*,*i*_ is the SE coupling tensor component. The meaning of eqn (3) is that **E**_**m**_ may not only produce a perturbation along its direction, but also perpendicular to it. For the sake of simplicity, only the diagonal terms of the SEC are considered, *i.e.*, the electric field does not modify the orientation of the principal axes, a circumstance theoretically verified for a Cu_3_ triangle.^23^

The effect of SEC on the exchange interaction can be correlated with the change in the electric dipole moment when an electron hops from one site to another, a virtual process responsible for the AFM exchange interaction.^58^ The perturbation Δ*J* on the exchange interaction was introduced as

4 *J*_*ln*,*E*_ = *J*_*ln*_ + (**p̂**_*ln*_·**Ê**_**m**_)Δ*J*

where **Ê**_**m**_ and **p̂**_*ln*_ are the unit vectors of the applied **E**_**m**_ and of the electric dipole moment that is associated with the spin flip that transforms the high spin state to a broken symmetry state.^24,58^ This dipole is assumed to lie in the plane of the triangle and perpendicular to the line connecting the Cr_*l*_ and Cr_*n*_ sites, as theoretically predicted for other molecular triangles^29,30^ with a deviation smaller than 10°.^24^ The same value of Δ*J* was considered for the three Cr–Cr pairs.

An electric field effect on DM interaction could also be present. It was proposed^59^ and recently confirmed by *ab initio* calculations^20^ that such an effect takes the form:

5 Δ**G**_*ln*_,_*E*_ ∝ (**E** × **r**_*ln*_)

where **r**_*ln*_ is the vector joining spin centers *l* and *n*. Despite the different forms of (4) and (5), an electric field applied in the plane of the triangle unbalances the values of the DM coupling constants but does not alter the average value, similar to what observed for *J*. However, such an asymmetry in the *G* values has no detectable effect on the effective *g* values, as shown in Fig. S17. Additionally, significant *G* values and related electric field effects are expected for metal ions showing close to first order SOC,^60^ which is not the case for Cr^III^.

To mimic the modulation of **E**_**m**_ used in the experiments, the EFM-EPR spectra were simulated as the difference between the absorption spectra acquired with +**E**_**m**_ and −**E**_**m**_. This treatment is valid in the approximation that the shift of the resonance field is much smaller than the signal linewidth.^11^ Since the first publications,^21,23^ the analysis of experimental data obtained by EFM-EPR on single crystals has reached a more mature stage, particularly in terms of simulation and interpretation of the resulting spectra, including a comprehensive analysis of molecular symmetry, line shape, and intensity.^16^ This development allows for the determination of the SE coupling tensor component *T*_*j*,*i*_ = ∂*g*_*i*_/∂*E*_*j*_, going therefore well above the mere comparison of the intensity of the EFM-EPR and EPR signals for the evaluation of the SEC.

Simulations of the EFM-EPR spectra with the phenomenological model are reported in the bottom panels of Fig. 6. The spectrum acquired with **a**//**B**_**0**_//**E**_**m**_ (panel a) was simulated with Δ*g*_*x*,*a*_ = Δ*g*_*y*,*a*_ = 1 × 10^−6^ (Δ*g*_*i*,*j*_ = *E*_*j*_*T*_*j*,*i*_). In this orientation, **E**_**m**_ can also influence *J* and, through the combination with the weak DM interaction, the resonance position. Indeed, the spectrum can be simulated with Δ*J* = 2 × 10^−6^ cm^−1^ (Δ*J*/*J* ∼ 10^−7^), and a reasonable simulation is also obtained by including both effects, as reported in Fig. S18. Disentangling the two contributions is a challenging task. However, when **E**_**m**_//c*, *i.e.*, orthogonal to the spin flip-induced dipole moment, only the SEC effect on *g* survives. For c*//**B**_**0**_//**E**_**m**_ (Fig. 6c) a satisfying simulation is obtained considering Δ*g*_*z*,*c*_ = 0.8 × 10^−6^. When c*//**E**_**m**_ but −**b**//**B**_**0**_ Δ*g*_*x*,*c*_ = Δ*g*_*y*,*c*_ = 0.9 × 10^−6^ needs to be included to get the correct intensity (Fig. 6d). Additionally, when **E**_**m**_ is along **a**, but the signal is acquired with c*//**B**_**0**_ (Fig. 6b), neither Δ*J* nor Δ*g*_*x*,*a*_ = Δ*g*_*y*,*a*_ = 1 × 10^−6^ are sufficient to reproduce the intensity of the EFM-EPR spectrum (see Fig. S19). Consequently, a term Δ*g*_*z*,*a*_ = 0.9 × 10^−6^ is introduced, which, however, does not alter the simulation of the spectrum acquired with **a**//**B**_**0**_//**E**_**m**_. As expected, both simulations, whether Δ*g* or Δ*J* is considered, yield zero EFM-EPR spectra for **b**//**E**_**m**_ (Fig. 6d), consistent with the experimental results. Therefore, we determined the first and third rows of the tensor 
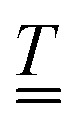
. The second row cannot be experimentally determined (n.d.) because for the given crystal symmetry of 1 the EFM-EPR signal is zero when the electric field is applied along **b**. The resulting SEC tensor is:6
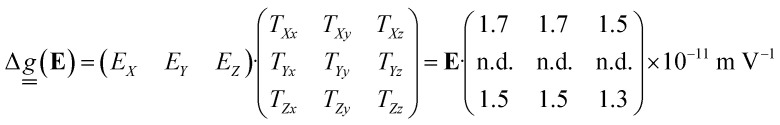


Two possible mechanisms can contribute to SEC on single-ion parameters: (i) atomic displacement under the electric field if the latter couples with a permanent electric dipole moment, (ii) a pure electronic one, which does not require an electric dipole moment, but is usually weaker.^15^ A SEC effect Δ*g*_*E*_/*E* of the order of 1–2 × 10^−11^ m V^−1^ is observed in Cr_3_ when the electric field is directed along the *a* or c* axes. This value is approximately one order of magnitude larger than that determined for a Cu_3_ triangle in directions lacking a permanent electric dipole, but it is smaller than the value along the Cu_3_ polar axis.^24^ Given the low symmetry of the Cr_3_ system, it is reasonable to observe an almost isotropic and not negligible SEC effect on the 
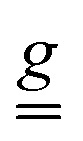
 tensor. The inclusion in the phenomenological model of the dipolar interaction between the Cr^III^ pairs does not alter the evaluation of the SEC (see Fig. S20).

The upper limit for SEC on *J*, 3 × 10^−11^ cm^−1^ m V^−1^, is more than two orders of magnitude smaller than that estimated by *ab initio* calculations and experimentally detected in the Cu_3_ triangle.^24^ Also, considering the weaker AFM interaction between Cr^III^ spins, the relative value Δ*J*_*E*_/*J* remains significantly smaller than in Cu_3_. This finding can be tentatively assigned to the different bridge of the latter, *i.e.*, the multi-chelating ligand anion of tris(2-hydroxybenzylidene)triaminoguanidine, which was found to contribute significantly to the spin-flip-induced dipole moment.^24^ Indeed, the relative SEC on *J* in Cr_3_ has the same order of magnitude as that computed for a Cu_3_ system in a polyoxometalate structure.^30^

## Conclusions

In this study, we synthesized an oxo-centered Cr_3_ frustrated spin triangle crystallizing in a non-centrosymmetric space group. A multi-technique approach was employed to fully characterize its spin Hamiltonian. In particular, the weak anisotropy induced by the presence of antisymmetric exchange was disentangled from the anisotropy of the 
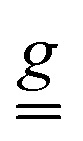
 tensor, by means of torque magnetometry in variable magnetic fields and temperatures, providing the essential foundation to investigate spin-electric effects in this system. To this purpose, we employed EPR spectroscopy under a modulated electric field. When properly applied to non-centrosymmetric crystals, EFM-EPR spectroscopy demonstrates exceptional sensitivity to electric-field-induced changes, enabling precise quantification of the effects through spectral simulations. The independent control of the magnetic and electric field orientations within the molecular reference frame further allows assessment of the anisotropy of the spin–electric effect and a microscopic disentanglement of its different contributions.

In the specific case of Cr_3_, which had previously shown no sizable spin–electric coupling, we were able to quantify the electric-field effect on the single-ion 
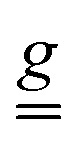
 tensor and to estimate an upper limit for the effect on the isotropic exchange interaction. The observation that the SEC on *J* is significantly smaller than that observed for a Cu_3_ complex bridged by an organic ligand, yet comparable to that computed for a Cu_3_ polyoxometallate, highlights the critical role of the bridging ligand in mediating the SEC effect.

Overall, these findings demonstrate the power of EFM-EPR spectroscopy as a quantitative probe of SEC in molecular systems. Building on its sensitivity and versatility, a systematic investigation of different bridging ligands, supported by theoretical modeling, could establish design criteria to optimize SEC effects, thereby paving the way for rational engineering of molecular systems with efficient electric control of spin.

## Author contributions

RS, MS, and MF conceptualized the idea of this work. BS synthesized and structurally characterized the material under the supervision of MS and TO. LT recorded the EPR spectra and performed the magnetic characterization under the supervision of MP and RS. AC, LT, and MF recorded the EFM-EPR spectra. AC simulated the EPR and EFM-EPR spectra under the supervision of MF. All authors have critically reviewed each aspect of the research and have contributed to the drafting and editing of the manuscript.

## Conflicts of interest

There are no conflicts to declare.

## Supplementary Material

SC-017-D5SC08012F-s001

SC-017-D5SC08012F-s002

## Data Availability

Experimental and simulated magnetic data and spectra will be available from the authors upon request. CCDC 2493370 contains the supplementary crystallographic data for this paper.^61^ Supplementary information (SI): additional figures; tables reporting crystallographic data (CCDC 2493370), and reference frame rotations; materials and methods. See DOI: https://doi.org/10.1039/d5sc08012f.
