# Supplementary material for: Sensitive detection of spin-electric coupling in a Cr3 antiferromagnetic triangle
Source: Chem Sci. 2025 Dec 4;17(6):3329–38. doi: 10.1039/d5sc08012f (PMC12720930; doi:10.1039/d5sc08012f)
Supplement: SC-017-D5SC08012F-s001 [file SC-017-D5SC08012F-s001.pdf]

Supplementary Information (SI) for Chemical Science.
This journal is © The Royal Society of Chemistry 2025

Supporting Information

Sensitive Detection of Spin-Electric Coupling in a Cr_3 Antiferromagnetic Triangle

Leonardo Tacconi,[a] Shubham Bisht,[b] Alberto Cini,[c,d] Mauro Perfetti,[a] Tomas Orlando,[e] Maria Fittipaldi,[c,d,*] Michael Shatruk,[b,*] Roberta Sessoli[a,d,*]

[a] *Dipartimento di Chimica "U. Schiff", Università degli Studi di Firenze, I-50019 Sesto Fiorentino, Firenze, Italy*

[b] *Department of Chemistry and Biochemistry, Florida State University, Tallahassee, FL 32306, USA*

[c] *Dipartimento di Fisica e Astronomia, Università degli Studi di Firenze, I-50019 Sesto Fiorentino, Firenze, Italy*

[d] *Consorzio Interuniversitario Nazionale per la Scienza e Tecnologia dei Materiali, I-50121 Firenze, Italy*

Contents:

Figure S1. FT-IR spectrum of the sample **1**·$6H_2O$ S2
Figure S2. TGA spectrum of the sample **1**·$6H_2O$ S2
Figure S3. Experimental and simulated χT data on powders of **1** S2
Figure S4. Temperature-dependent susceptibility data on oriented crystal of **1** S3
Figure S5. Field-dependent susceptibility data on the oriented crystal of **1**. S3
Figure S6. Orientational Representation of **1** for CTM measurements S4
Figure S7. CTM data on **1** for rotation 2 .... S4
Figure S8. Reference frames of the ***g*** and ***D*** tensors for triangle 1 .... S5
Figure S9. Simulated CTM data for rotation 1 by using g-anisotropy only S5
Figure S10. Simulated CTM data for rotation 1 by using DM-anisotropy only S6
Figure S11. Simulated Magnetization curves on single crystals of **1** S7
Figure S12. Simulated χT data on single crystals of **1** S7
Figure S13. Simulated magnetic properties by including the dipolar interactions S8
Figure S14. EPR spectra acquired on the single crystal of **1** S9
Figure S15. Gaussian distribution of δJ values used to simulate the EPR spectra S10
Figure S16. EFM-EPR Spectrum of **1** with ***a*** crystallographic axis parallel to $\mathbf{B_0}$ and -$\mathbf{E_m}$ S11
Figure S17. Zeeman diagrams computed assuming an E field effect on *J* and *G*. S12
Figure S18. EFM-EPR Spectrum of **1** with ***a*** crystallographic axis parallel to $\mathbf{B_0}$ and $\mathbf{E_m}$ S13
Figure S19. EFM-EPR Spectrum of **1** with ***c***** crystallographic axis parallel to $\mathbf{B_0}$ and ***a*** parallel to $\mathbf{E_m}$ S13
Figure S20. EPR and EFM-EPR simulations with inclusion of dipolar interaction S14
Table S1. Data collection and structure refinement parameters for **1** S15
Table S2. Euler angles describing rotation from crystallographic to molecular reference frame S16
Table S3. Euler angles describing rotation from molecular to tensor reference frame S16
Material and Methods S16

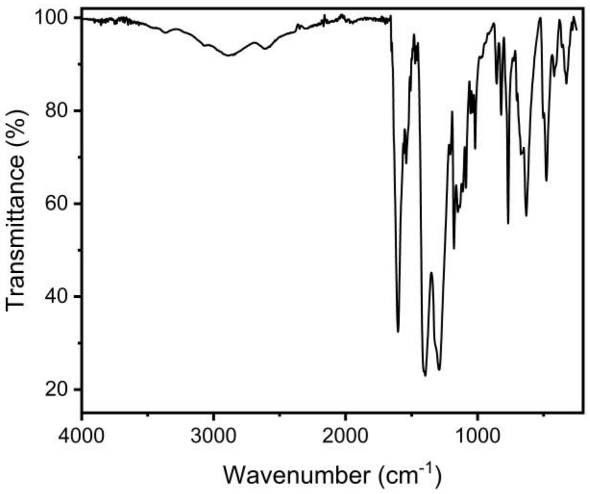

Figure S1. The FT-IR spectrum of complex **1**·$6H_2O$.

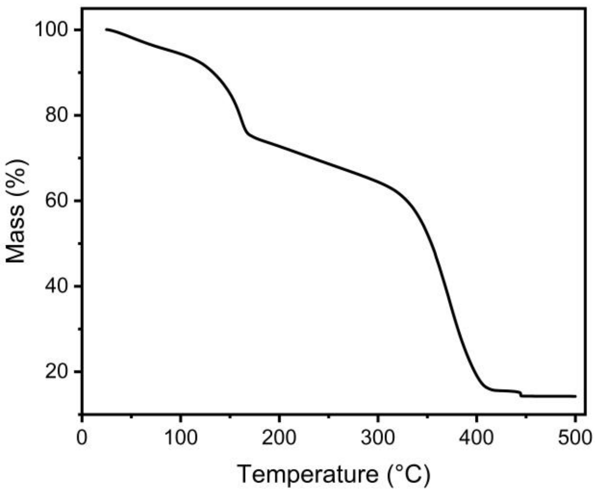

Figure S2. The TGA curve of the sample **1**·$6H_2O$.

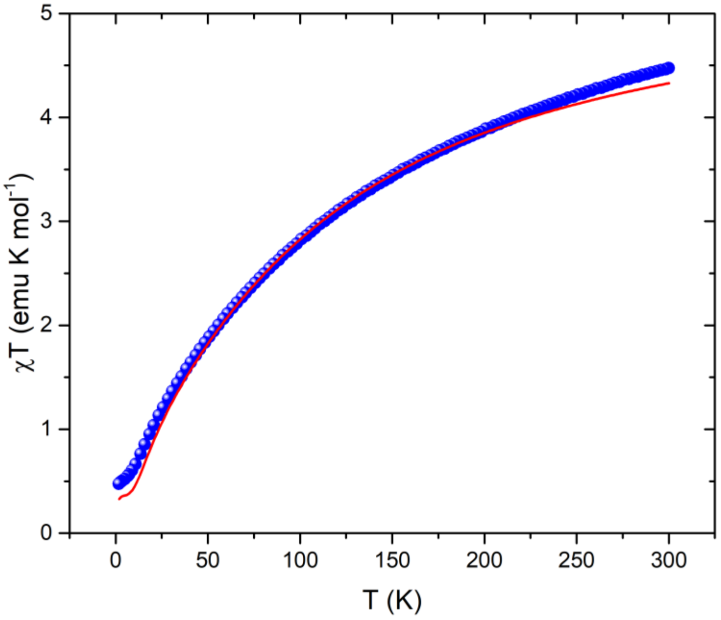

Figure S3. Experimental (dots) and simulated (line) χT curve at 10 kOe on powders of **1**. Simulations were obtained using the Hamiltonian parameters reported in the main text.

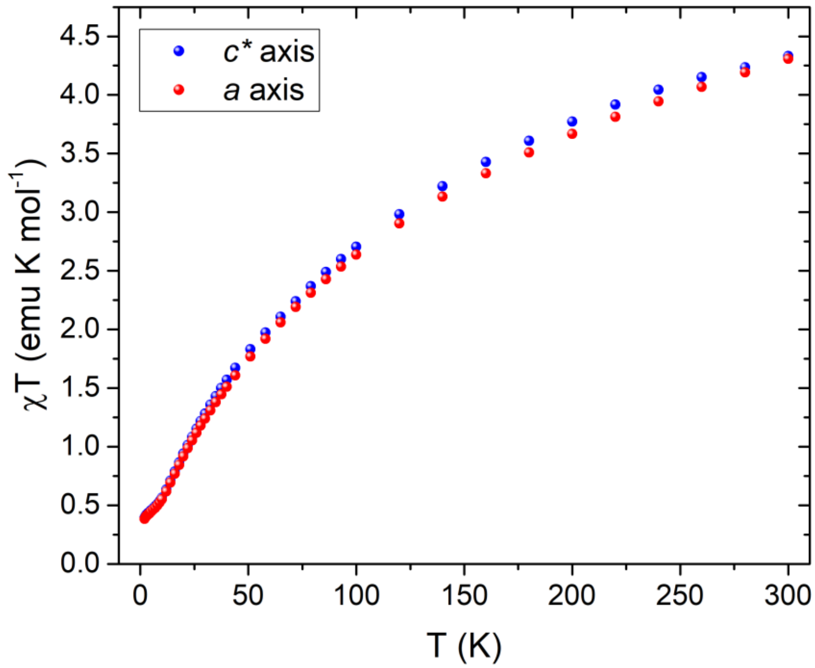

Figure S4. Temperature dependence of χT for an oriented single crystal of **1** measured under an applied magnetic field of 10 kOe. The magnetic field was applied along the crystallographic *a* axis (red dots) and *c** axis (blue dots).

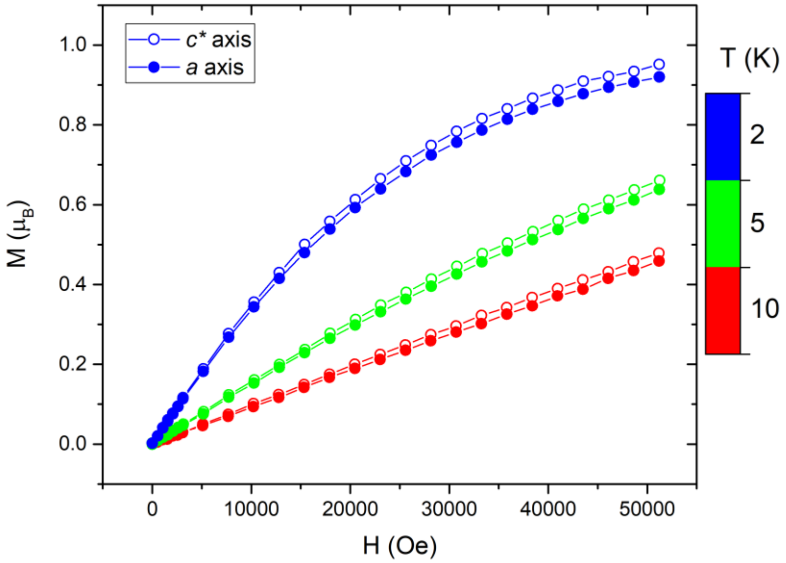

Figure S5. Magnetic field dependence of the magnetization curves of **1** acquired at different temperatures and with the field applied parallel to two different crystallographic orientations.

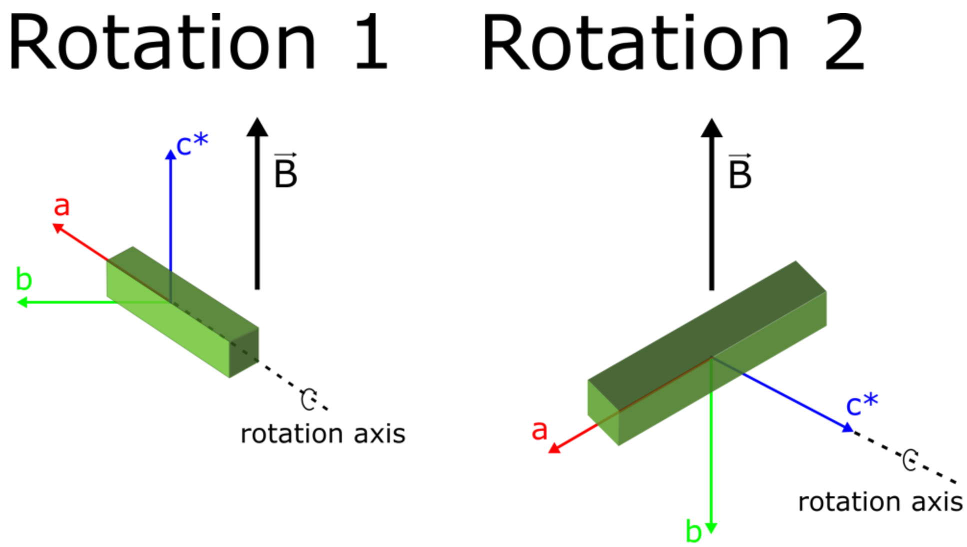

Figure S6. Representation of the two rotations performed during cantilever torque magnetometry experiments with respect to the crystallographic *abc** reference frame. The situation depicted in the image refers to the rotation angle being equal to 0°.

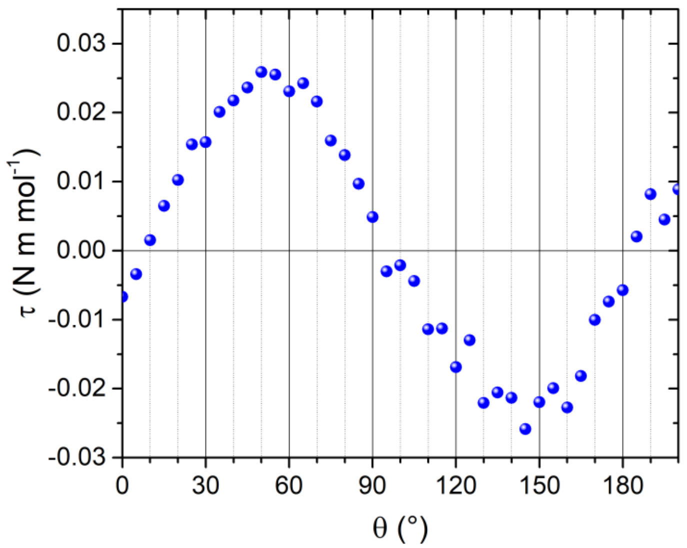

Figure S7. Experimental (dots) cantilever torque magnetometry curve acquired on **1** during *Rotation 2* (along ***c****) at 2K and 9T.

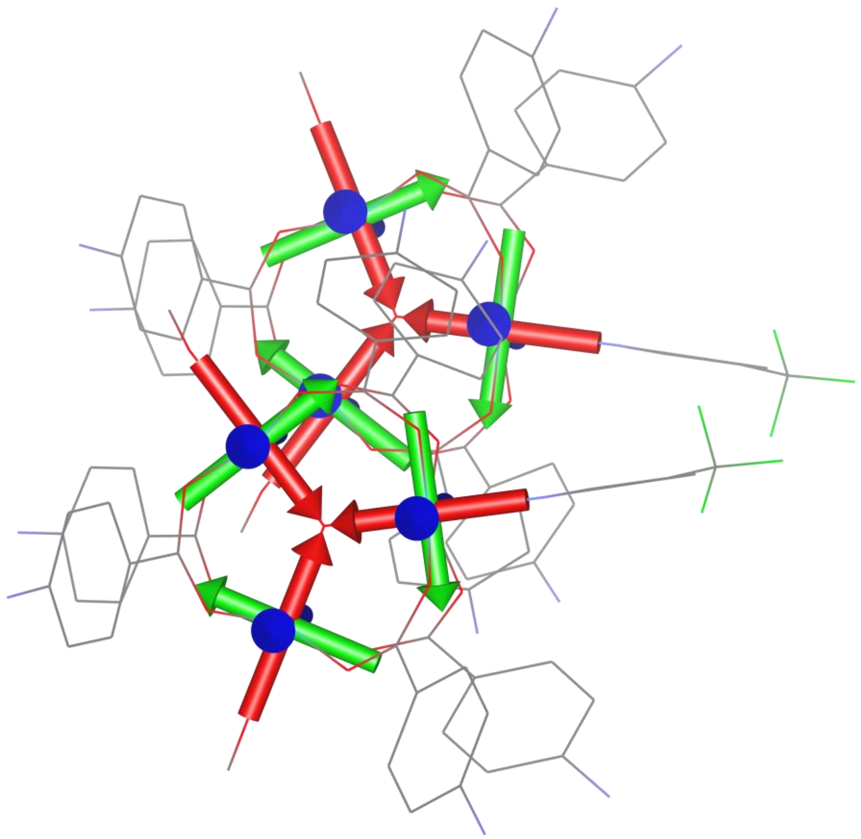

Figure S8. Reference frames of the ***g*** and ***D*** tensors for the two triangles, obtained from Euler matrices in Tables S2-S3. The axes were calculated considering the Euler angles reported in Tables S2-S3. Axes color code: red = *x*, green = *y*, blue = *z*. Atoms color code: Cr, sky blue; O, red; N, blue; C, grey; F, green.

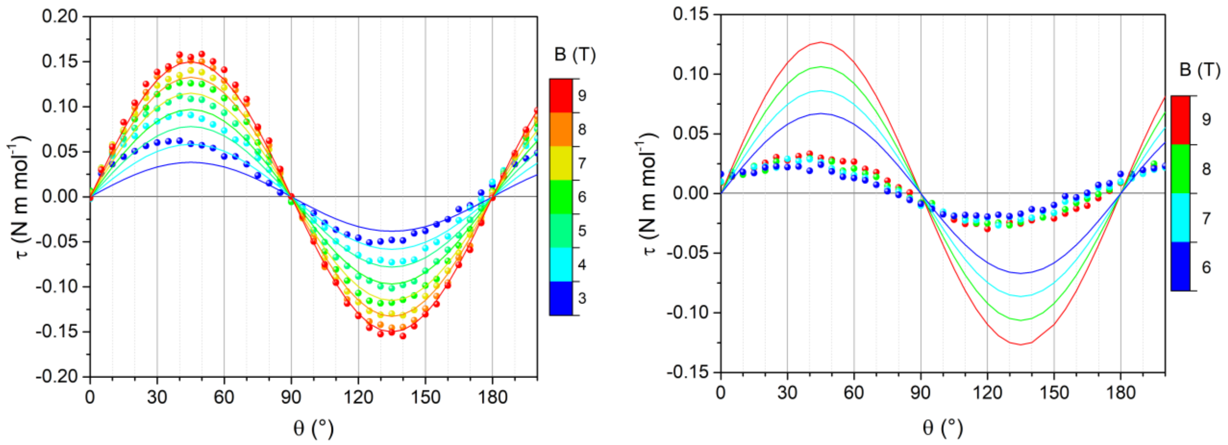

Figure S9. Experimental (dots) and simulated (lines) cantilever torque magnetometry curves acquired on **1** during *Rotation 1* at 2K (left) and 5K (right) and different magnetic fields. The simulated curves were calculated considering only an anisotropy of the *g* factor, as discussed in the main text.

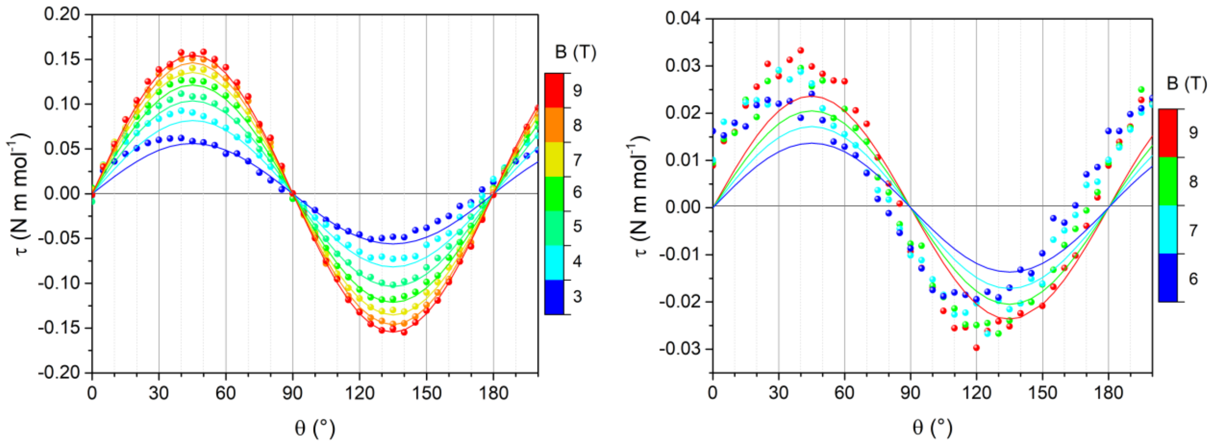

Figure S10. Experimental (dots) and simulated (lines) cantilever torque magnetometry curves acquired on **1** during *Rotation 1* at 2K (left) and 5K (right) and different magnetic fields. The simulated curves were calculated considering an anisotropy induced only by the Dzyaloshinskii–Moriya interaction, as discussed in the main text.

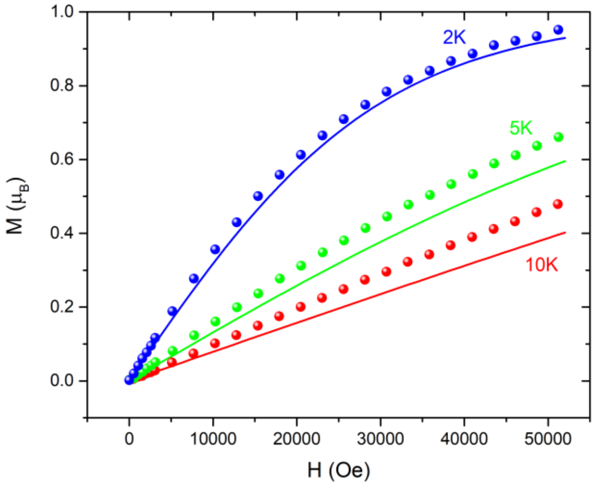

Figure S11. Experimental (dots) and simulated (lines) magnetization curves at different curves on single crystals of **1**, with the magnetic field applied along the *c** (left) and *a* (bottom) axes. Simulations were obtained using the Hamiltonian parameters reported in the main text.

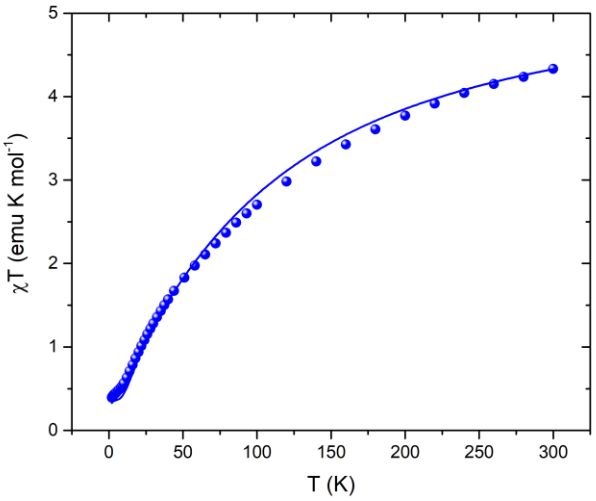

Figure S12. Experimental (dots) and simulated (lines) χT curves at 10 kOe on single crystals of **1**, with the magnetic field applied along the *c** (left) and *a* (bottom) axes. Simulations were obtained using the Hamiltonian parameters reported in the main text.

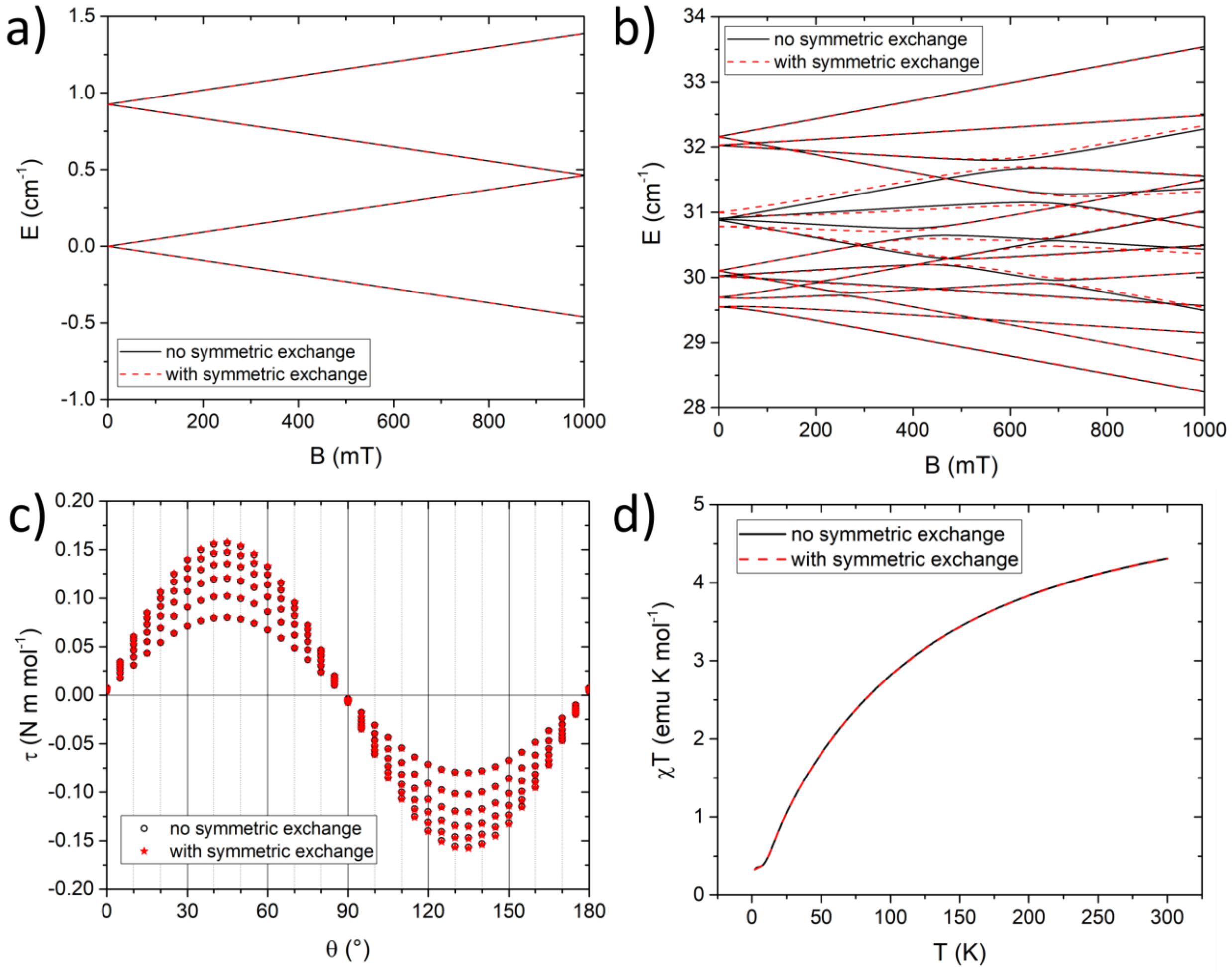

Figure S13. a,b) Computed Zeeman diagrams along the *z*-axis neglecting (solid black) and including (dashed red) dipolar interactions, contributing to the symmetric (or anisotropic) part of the exchange. The same comparison is reported for the computed torque data at 2 K (c) and the powder-averaged magnetic susceptibility (d). The main components of the symmetric exchange tensor D_{ij} were calculated from the anisotropy of the g tensors and are $D_{12} = [-0.0939, 0.0469, 0.0470]$, $D_{31} = [-0.0958, 0.0479, 0.0479]$ and $D_{23} = [-0.0951, 0.0475, 0.0476]$, expressed in wavenumbers.

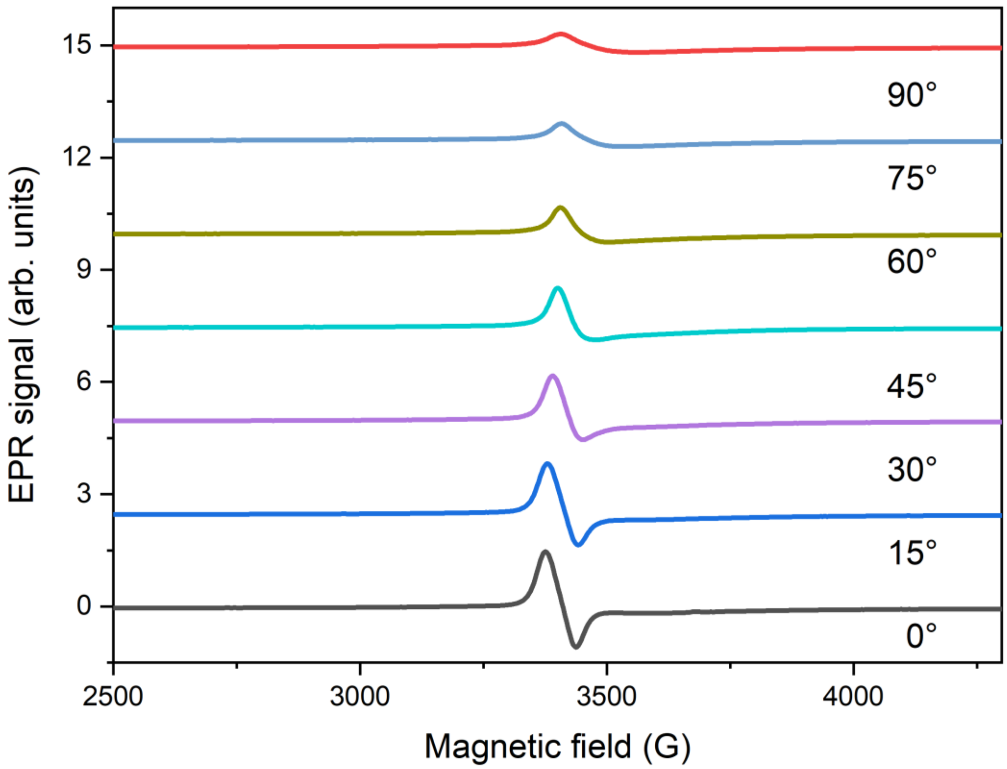

Figure S14. EPR spectra acquired on the single crystal of **1** in steps of 15° from $\boldsymbol{c}^*//\mathbf{B}_0$ (black line) to $\boldsymbol{a}//\mathbf{B}_0$.

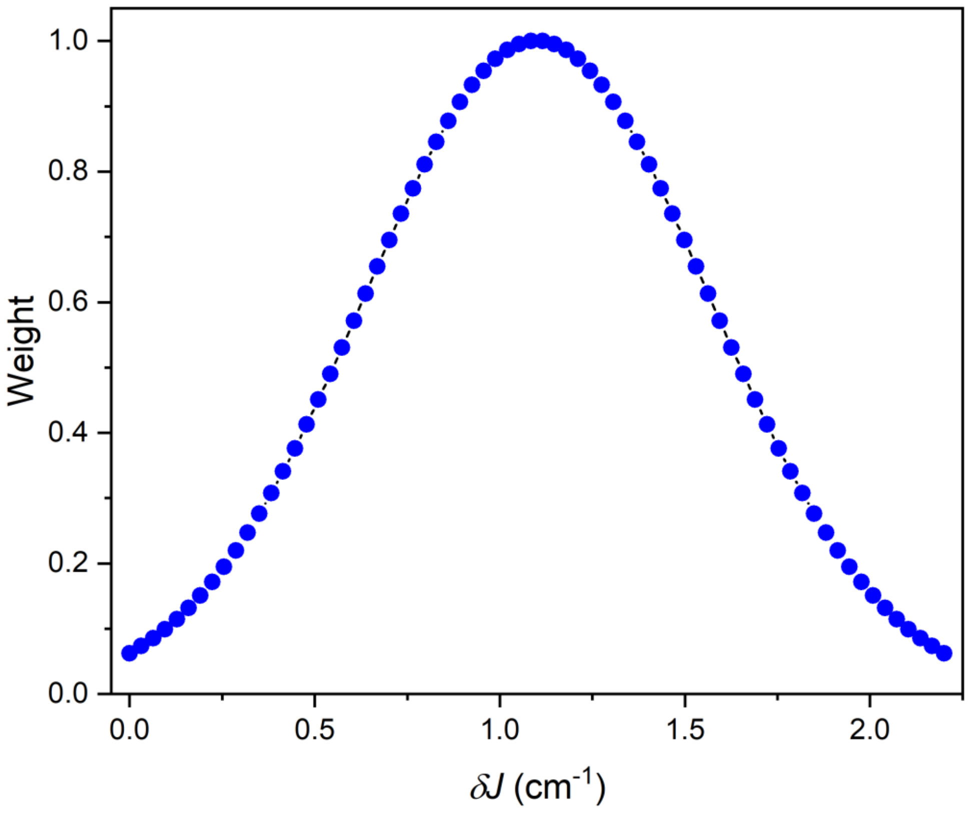

Figure S15. Gaussian distribution of δJ values used to simulate the EPR spectra.

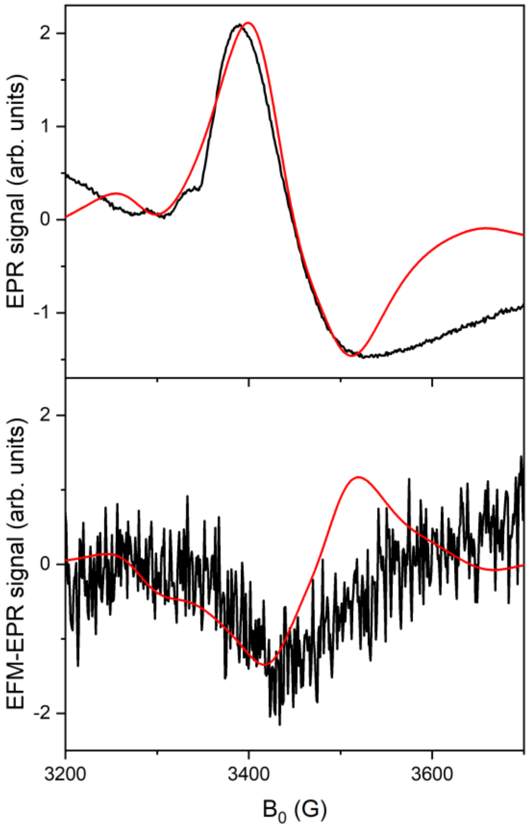

Figure S16. EPR spectrum (top panel) and corresponding EFM-EPR spectrum (bottom panel) acquired on a single crystal of **1** at 15 K with the ***a*** crystallographic axis parallel to $\mathbf{B_0}$ and -$\mathbf{E_m}$. The EFM-EPR spectrum intensity is rescaled by the number of acquisitions *n* = 14. Red lines are the simulations.

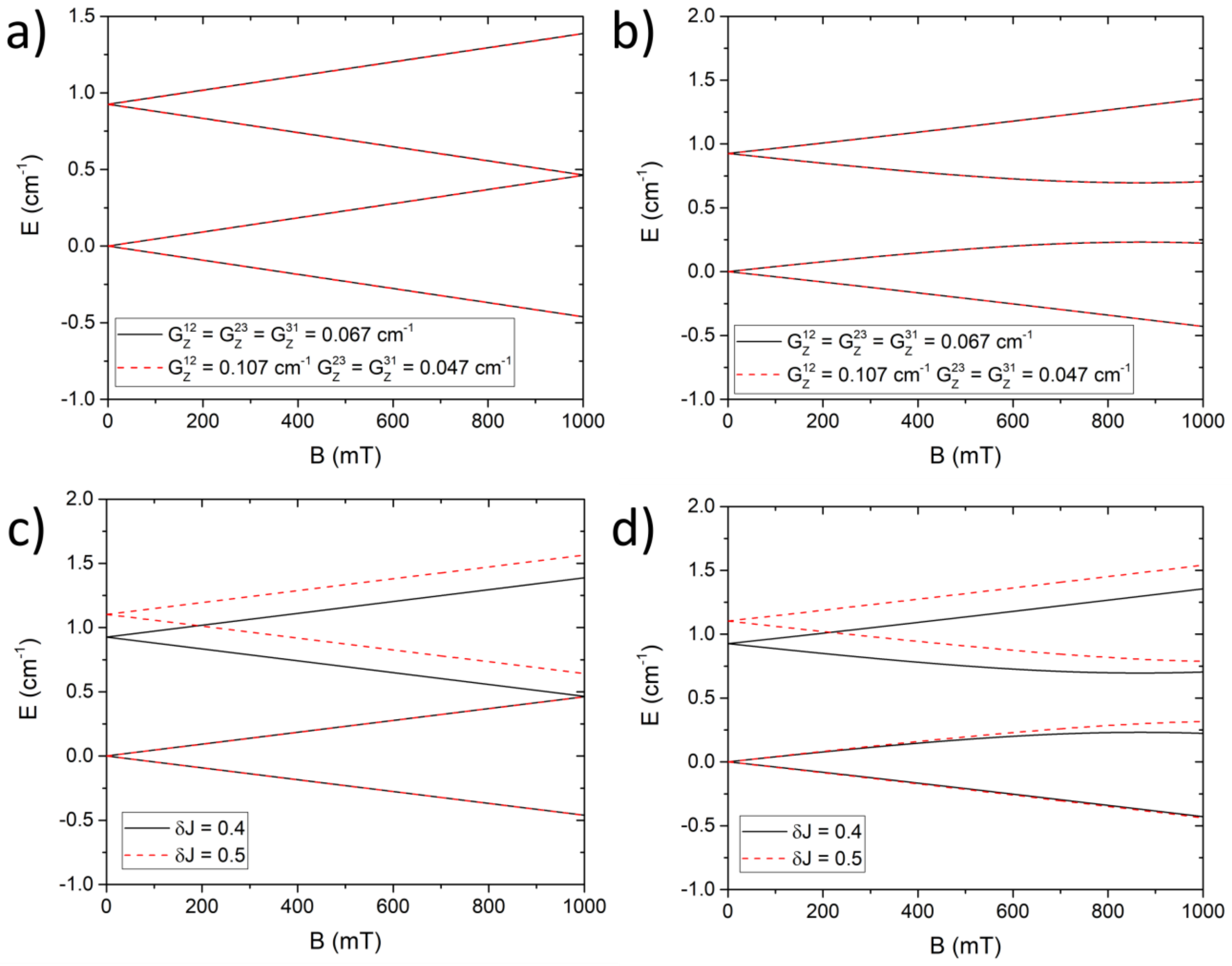

Figure S17. a,b) Computed Zeeman diagrams assuming an electric field effect on *G* according to eq. 5 of the main text with the magnetic field applied perpendicular to the Cr_3 plane and parallel to it, respectively. c,d) The same, assuming an electric field effect on *J* according to eq. 4 of the main text.

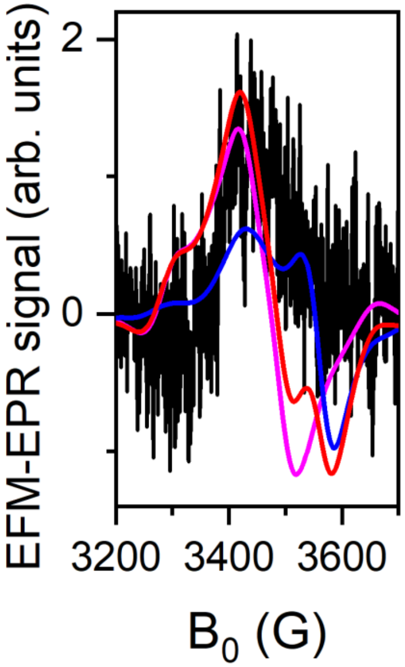

Figure S18. EFM-EPR spectrum acquired on a single crystal of **1** at 15 K with the ***a*** crystallographic axis parallel to $\mathbf{B_0}$ and $\mathbf{E_m}$. The blue line is the simulation considering a perturbation of $\Delta J = 2\times10^{-11}$, the magenta line corresponds to the simulation with $\Delta g_{x,a} = \Delta g_{y,a} = 1 \times 10^{-6}$, while the red line includes both contributions.

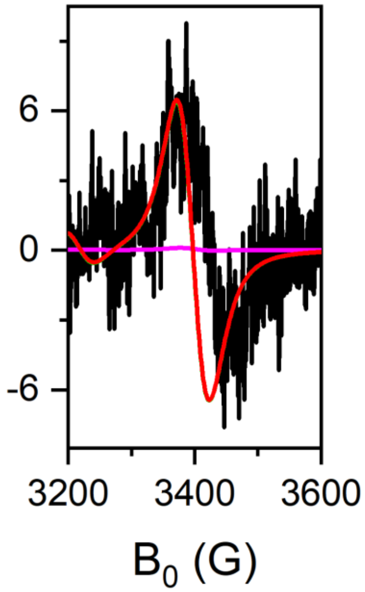

Figure S19. EFM-EPR spectrum acquired on a single crystal of **1** at 15 K with the ***c**** crystallographic axis parallel to $\mathbf{B_0}$ and ***a*** parallel to $\mathbf{E_m}$. Simulations considering a a perturbation on the *g* tensor as described by Eq. 3 in main are reported: simulation with $\Delta g_{x,a} = \Delta g_{y,a} = 1 \times 10^{-6}$ (magenta line), simulation with $\Delta g_{z,a} = 0.9 \times 10^{-6}$ (green line) and simulation with $\Delta g_{x,a} = \Delta g_{y,a} = 1 \times 10^{-6}$ and $\Delta g_{z,a} = 0.9 \times 10^{-6}$ (red line). The latter two are indistinguishable.

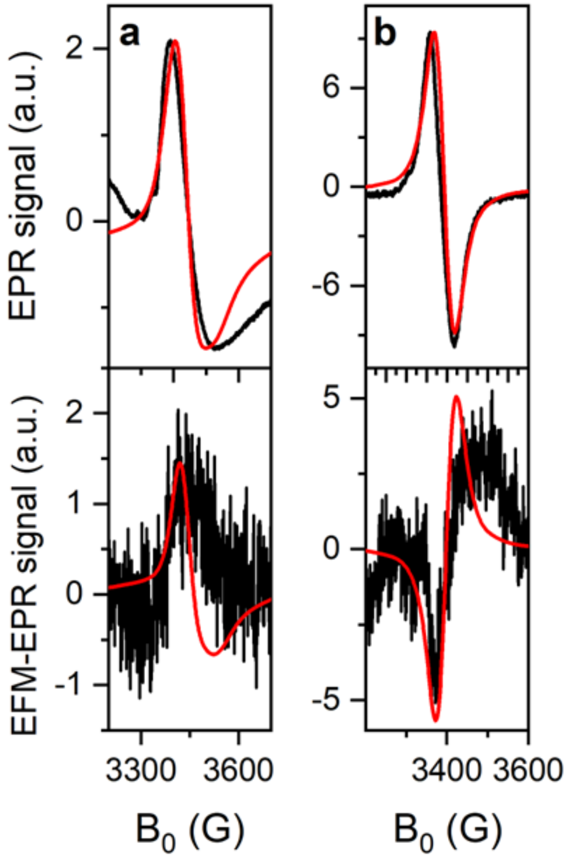

Figure S20. EPR and EFM-EPR spectra acquired on a single crystal of **1** at 15 K with: (**a**) the $\boldsymbol{a}$ crystallographic axis parallel to $\mathbf{B_0}$ and $\mathbf{E_m}$; (**b**) the $\boldsymbol{c^*}$ crystallographic axis parallel to $\mathbf{B_0}$ and $\mathbf{E_m}$. In the simulations, the dipolar interaction between each Cr(III) couple is considered with values $dip_{12} = [-0.0939, 0.0469, 0.0470]$ cm^{-1}, $dip_{31} = [-0.0958, 0.0479, 0.0479]$ cm^{-1}, and $dip_{23} = [-0.0951, 0.0475, 0.0476]$ cm^{-1}. The orientation of the dipolar axes for D_{13} is defined by Euler angles ([329.9° 0 0] for both triangles with respect to the molecular frame, the rotation is intended as an intrinsic ZY′Z′′ rotation). For the simulation of the EFM-EPR spectra, a perturbation on the g tensor as described by Eq. 3 is considered with the values of the $\Delta g_{i,j}$ already reported in the manuscript.

SUPPORTING TABLES

Table S1. Data collection and structure refinement parameters for **1**.

Compound	$Cr_3F_3O_{50}N_{14}C_{64}H_{108}$ (**1**·14MeOH)[a]
Temperature, K	200.0(4) K
CCDC number	2493370
Formula weight	2086.64
Space group	*Cc*
a, Å	13.0818(1)
b, Å	26.5227(2)
c, Å	28.5781(2)
α, deg	90
β, deg	95.153(1)
γ, deg	90
V, $Å^3$	9875.5(1)
Z	4
Crystal color	dark-green
Crystal size, mm^3	0.72×0.37×0.30
d_{calc}, g cm^{-3}	1.403
□, mm^{-1}	3.598
□, Å	1.54184
2$□_{max}$, deg	153.408
Total reflections (R_{int})	178672 (0.059)
Unique reflections	19685
Parameters refined	979
Restraints used	15
R_1, wR_2 [I > 2□(I)][b]	0.068, 0.189
R_1, wR_2 (all data)	0.075, 0.197
Goodness of fit[c]	1.036
Diff. peak/hole, e $Å^{-3}$	0.44, –0.40
Flack Parameter	0.080(6)

[a] All but one of the MeOH molecules have been assigned to the formula based on the solvent mask procedure implemented in the Olex software.

[a] $R_1 = \Sigma||F_o|-|F_c||/\Sigma|F_o|$; $wR_2 = [\Sigma[w(F_o^2-F_c^2)^2]/\Sigma[w(F_o^2)^2]]^{1/2}$;

[b] Goodness-of-fit = $[\Sigma[w(F_o^2-F_c^2)^2]/(N_{obs}-N_{params})]^{1/2}$, based on all data.

Table S2. Interatomic distances and bond angles in the crystal structure of **1**.

Bond Lengths (Å)	Cr(1)	Cr(2)	Cr(3)
Cr–(μ_3-O)	1.908(4)	1.894(4)	1.896(4)
Cr–O_{MeOH} (or –N_{pyr})	2.048(4)	2.078(5)	2.111(5)
Cr–O_{BzO}	1.955(5)	1.995(4)	1.963(5)
	1.936(5)	1.927(5)	1.947(4)
	1.977(5)	1.947(5)	2.000(5)
	1.994(5)	1.995(5)	2.000(5)

Cr–Cr Distances (Å)		Cr–O–Cr Angles (°)	
Cr(1)–Cr(2)	3.301(1)	Cr(1)–O–Cr(2)	120.4(2)
Cr(1)–Cr(3)	3.279(1)	Cr(1)–O–Cr(3)	119.0(2)
Cr(2)–Cr(3)	3.289(1)	Cr(2)–O–Cr(3)	120.4(2)

Table S2 – Euler angles describing the rotation from the crystallographic to the molecular reference frame. The rotation is intended as an intrinsic $ZY'Z''$ rotation.

	$\hat{R}_{C-M}(\psi,\xi,\rho)$		
	$\psi(°)$	$\xi(°)$	$\rho(°)$
**Triangle 1**	152.960	10.632	19.490
**Triangle 2**	207.039	10.632	160.509

Table S3 – Euler angles describing the rotation from the molecular to the tensors reference frame. The rotation is intended as an intrinsic $ZY'Z''$ rotation

		$\hat{R}_{M-T}(\psi,\xi,\rho)$		
		$\psi(°)$	$\xi(°)$	$\rho(°)$
**Triangle 1**	**Spin 1**	179.833	0.334	179.999
	**Spin 2**	299.833	0.334	179.999
	**Spin 3**	59.833	0.334	179.999
**Triangle 2**	**Spin 1**	0.166	0.334	180.000
	**Spin 2**	120.166	0.334	180.000
	**Spin 3**	240.166	0.334	180.000

Materials and Methods

All reactions were performed under aerobic conditions. The starting materials, $Cr(NO_3)_3 \cdot 9H_2O$ (99.0%, Millipore Sigma), 4-aminobenzoic acid (4-H_2NBzOH, >99%, Beantown Chemical), 3-trifluoromethylpyridine (3-F_3Cpy, 99.0%, Ambeed), methanol (MeOH, >99.0%, Fisher Scientific), and diethyl ether (Et_2O, >98%, VWR), were used as received. Elemental analyses were performed by Atlantic Microlab, Inc. (Norcross, GA, USA).

[Cr_3(μ_3-O)(μ_2-4-H_3NBzO)$_6$(3-CF_3py)(MeOH)$_2$](NO_3)$_7$ (1). A solution of 4-H_2NBzOH (411 mg, 3.00 mmol) in 5 mL of MeOH was added dropwise to a solution of $Cr(NO_3)_3 \cdot 9H_2O$(600 mg, 1.50 mmol) in 10 mL of MeOH. The resultant green solution was refluxed for 5 h, concentrated to a half of the initial volume, and filtered. To the filtrate, 3-F_3Cpy (600 µL, 5.0 mmol) was added. The solution was refluxed for 12 h, cooled to room temperature, and filtered. Vapor diffusion of Et_2O into the filtrate resulted in crystallization of **1**. Yield = 470 mg (60%). *Elem. Anal.* (%): Calcd (Found) for $Cr_3F_3N_{14}O_{42}C_{50}H_{66}$ (**1** · $6H_2O$): C, 34.35 (34.04); H, 3.81 (3.64); N, 11.22 (11.25); F, 3.26 (3.48).

Physical Measurements. Fourier-transform infrared (FT-IR) spectra were recorded on a JASCO 6800 FT-IR spectrometer in the 4000−250 cm^{-1} range using a universal attenuated total-reflection accessory. Thermogravimetric analysis (TGA) was performed on a TGA-550 analyzer (TA Instruments) in the ranges from 24 to 500 °C at 10 °C /min under a continuous flow of argon gas.

X-Ray Crystallography. Single-crystal X-ray diffraction was performed on a Rigaku-Oxford Diffraction Synergy-S diffractometer using a monochromated Cu-Kα (λ =1.54184 Å) radiation source. A chosen single crystal was suspended in STP 65148 oil, mounted on a cryoloop, and cooled to the desired temperature under an N2 cold stream. The data set was recorded as ω-scans at 0.5° step width and integrated with the CrysAlis software package. An empirical absorption correction was applied using the SCALE3 ABSPACK algorithm. The space group was determined with CrysAlis, and the crystal structure solution and refinement were carried out with SHELX[1] using the interface provided by Olex2.[2] The final refinement was performed with anisotropic atomic displacement parameters for all non-hydrogen atoms, except for the atoms of some severely disordered nitrate ions which were treated isotropically. All H atoms were placed in calculated positions and refined in the riding model. In addition, a substantial disordered electron density, found in two voids of the crystal structure, was treated by the solvent mask procedure in Olex.[2] Full details of the crystal structure refinement and the final structural parameters have been deposited with the Cambridge Crystallographic Data Centre (CCDC). The CCDC registry number and a summary of data collection and refinement are provided in Table S1.

Magnetic Measurements. Magnetic measurements on powders and single crystals of **1** were performed using a magnetic property measurement system MPMS-3 (Quantum Design). The polycrystalline sample was loaded in a polycarbonate capsule and inserted in a plastic straw attached to a sample transport rod. Oriented single crystals were mounted inside a plastic straw using a minimal amount of grease to ensure stability. The temperature dependence of magnetic susceptibility of powders was measured under an applied magnetic field of 1 kOe in the range from 300 K to 1.8 K, while for single crystals, the measurements were carried out in an applied

magnetic field of 10 kOe. The data were corrected for diamagnetism from the sample holder and for intrinsic diamagnetism using tabulated constants.[3]

Cantilever Torque Magnetometry. Torque measurements were carried out on a Torque Magnetometry insert of a Quantum Design PPMS. A single-crystal sample of **1** was measured in a wide range of temperatures (2-250 K) and magnetic fields (2-9 T). During each measurement, the crystal was rotated by 180° around an axis perpendicular to the external magnetic field.

Electron Paramagnetic Resonance. X-band EPR measurements were performed using a 9 GHz Elexys E500 instrument (Bruker) equipped with a microwave frequency counter. An Oxford Instruments ESR900 continuous He flow cryostat was used to achieve low temperatures. An ER4122SHQE EPR resonator (Bruker) was used for the measurements. EPR spectra were acquired with a magnetic field modulation amplitude of 5 G, frequency of 100 kHz, a microwave power of 2.2 mW, a time constant of 20.48 ms, and a conversion time of 40.96 ms for the 2048 points field sweep, resulting in an acquisition time of 84 s.

The EFM-EPR experiments were performed with the same setup used for EPR, except for a modified sample holder. 19 A 30 kHz oscillating electric field of 60 kV/m was used to feed the electrodes. Given the heat load associated with this modulating Em, the EFM-EPR and the corresponding EPR spectra used as references were acquired at 15 K. To prevent discharge, the EPR tube, which housed the electrodes and sample, was filled with He gas and sealed. The EFM-EPR spectra were acquired with a microwave power of 68 mW, a time constant of 327.68 ms, and a conversion time of 655.36 ms for the 512-point field sweep, resulting in an acquisition time of 336 s for each spectrum.

Simulations of Magnetic Properties. DC, CTM, EPR, and EFM-EPR properties were simulated using custom MATLAB scripts based on EasySpin v6.[4]

References:

1. Sheldrick, G. M. Crystal structure refinement with SHELXL. *Acta Crystallogr. Sect. C* **2015**, *71*, 3-8; https://doi.org/10.1107/s2053229614024218.
2. Dolomanov, O. V.; Bourhis, L. J.; Gildea, R. J.; Howard, J. A. K.; Puschmann, H. OLEX2: a complete structure solution, refinement and analysis program. *J. Appl. Cryst.* **2009**, *42*, 339-341; https://doi.org/10.1107/s0021889808042726.
3. Bain, G. A.; Berry, J. F. Diamagnetic corrections and Pascal's constants. *J. Chem. Educ.* **2008**, *85*, 532-536; https://doi.org/10.1021/ed085p532.
4. Stoll, S.; Schweiger, A. EasySpin, a comprehensive software package for spectral simulation and analysis in EPR, *J. Magn. Reson.*, **2006**, *178*, 42-55; https://doi.org/10.1016/j.jmr.2005.08.013.
